# Viral load dynamics of SARS-CoV-2 Delta and Omicron variants following multiple vaccine doses and previous infection

**DOI:** 10.1038/s41467-022-33096-0

**Published:** 2022-11-07

**Authors:** Yonatan Woodbridge, Sharon Amit, Amit Huppert, Naama M. Kopelman

**Affiliations:** 1 The Gertner Institute for Epidemiology & Health Policy Research, Sheba Medical Center, Ramat Gan, Israel; 2 Department of Computer Science, Holon Institute of Technology, Holon, Israel; 3 Clinical Microbiology, Sheba Medical Center, Ramat Gan, Israel; 4 Sackler Faculty of Medicine, Tel-Aviv University, Tel-Aviv, Israel

**Keywords:** Viral infection, SARS-CoV-2, RNA vaccines, Epidemiology

## Abstract

An important aspect of vaccine effectiveness is its impact on pathogen transmissibility, harboring major implications for public health policies. As viral load is a prominent factor affecting infectivity, its laboratory surrogate, qRT-PCR cycle threshold (Ct), can be used to investigate the infectivity-related component of vaccine effectiveness. While vaccine waning has previously been observed for viral load during the Delta wave, less is known regarding how Omicron viral load is affected by vaccination status, and whether vaccine-derived and natural infection protection are sustained. By analyzing results of more than 460,000 individuals, we show that while recent vaccination reduces Omicron viral load, its effect wanes rapidly. In contrast, a significantly slower waning rate is demonstrated for recovered COVID-19 individuals. Thus, while the vaccine is effective in decreasing morbidity and mortality, its relatively small effect on transmissibility of Omicron (as measured here by Ct) and its rapid waning call for reassessment of future booster campaigns.

## Abstract (web summary)

COVID vaccination can reduce virus levels in breakthrough infections, which in turn may reduce transmission of the virus. By using qRT-PCR cycle threshold as a surrogate of virus levels, the authors here show that this positive effect of vaccination wanes relatively quickly for Omicron breakthrough infection.

## Introduction

Vaccine effectiveness is usually measured as the protection from infection, clinical disease or death^1,2^. However, this definition neglects an important aspect—the potential risk of transmission given infection. The latter has major implications on devising public health policies to reduce the spread of the pathogen. A prominent factor affecting infectivity is viral load (VL), which negatively correlates with the cycle threshold (Ct) values of quantitative real-time polymerase chain reaction (qRT-PCR). Thus, Ct values of routine diagnostic SARS-CoV-2 PCR tests are a readily available surrogate allowing clinicians and researchers to estimate infectiousness^3,4^.

In Israel, the BNT162b2 vaccination campaign was launched on December 19th, 2020. In light of a resurgence caused by the Delta variant, a third dose (“booster”) campaign was launched on July 29th, 2021, for individuals who had received the second dose at least 5 months earlier. By the end of January 2022, about 6.1 million people received two doses, out of which ~4.4 million people received three doses^5^. Nonetheless, Israel, like the rest of the world, experienced an unprecedented Omicron resurgence starting from mid-December 2021. In order to protect the older adults’ population, a fourth dose was administered to people over 60, high-risk individuals, and healthcare workers, starting from January 3rd 2022. Consequently, out of those who received three doses, ~650,000 have received four doses at the time of data extraction and analysis^5^.

In this retrospective study, we combined the comprehensive Israeli national vaccination data with Ct data of four laboratories performing SARS-CoV-2 PCR tests. Studies on the Delta variant have shown that vaccinated individuals have higher Ct (hence, lower VL), thus considered less infective, and that this effect wanes as time elapses^6,7^. We augment these studies by examining Omicron Ct in relation to vaccination and recovery statuses, and how Ct changes with time since vaccination/infection using nationwide qRT-PCR data. Notably, the waning effect of infection-induced protection has not been thoroughly analyzed before in terms of Ct and infectivity. We further compare Ct levels of individuals vaccinated with 2,3, or 4 doses vs. COVID-19 recovered individuals.

## Results

Ct data of positive tests were obtained from four molecular labs, two of which are major Israeli Health Maintenance Organization labs, representing together ~40% of the Israeli population, and the other two are labs commissioned to perform tests for the Israeli Ministry of Health (MoH). All of the PCR tests included in this study were part of MoH surveillance scheme, and charge-free for the consumer. We analyzed Ct values dating June 15, 2021–January 29, 2022, divided into two periods of Delta and Omicron dominance (see Methods & Supplementary Fig. 1 for further details). Separate analyses were conducted for the viral nucleocapsid gene (N, 315,111 measurements) and envelope gene (E, 228,125 measurements). The patterns observed for E were similar to those of N (see Supplementary Table 2, Supplementary Table 3, and Supplementary Figs. 2 and 3). To circumvent platform and other methodological variances between laboratories, each lab dataset was analyzed both separately and combined, with similar patterns observed (see Supplementary Information).

**Fig. 1:**
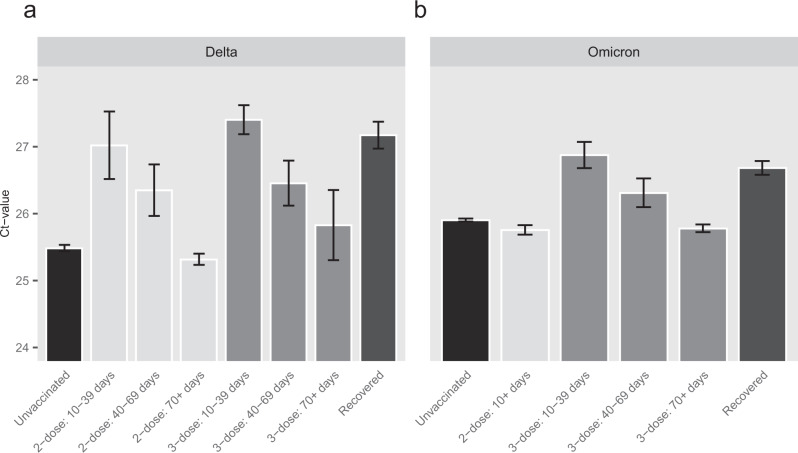
Ct values of the gene N. Adjusted Ct values of different vaccination statuses, measured by four laboratories, for the Delta (**a**, *n* = 101,897 independent samples), and Omicron (**b**, *n* = 181,634 independent samples) variants. Means were obtained from the weighted sum of age, sex and calendar time, and the reference group (see Supplementary Table 2). Error bars represent 95% CI’s around the means, obtained by using the estimated distribution of all four labs together (see Methods).

We first performed multivariate linear regression analysis on Ct values of each variant with vaccination status, laboratory, age, sex and calendar time (7-days bins) as covariates (see Supplementary Table 2). Vaccination status was defined as unvaccinated, 2-dose (divided to 3 bins, 10–39 days, 40–69, >70 days post-vaccination), 3-dose (divided to 3 bins, 10–39, 40–69, >70 days), 4-dose, or recovered who have not received any vaccination between the two infection events. Figure 1 depicts the adjusted Ct values for Delta and Omicron (see Supplementary Fig. 2 for extended results for each lab separately). Due to the small number of individuals in the 2-dose (early) groups for the Omicron period, all the 2-dose groups were combined.

Like previously reported^7,8^, during the Delta surge, 2-dose noticeably decreases VL (Fig. 1a), as seen by the decrease between the unvaccinated Ct level and that of the early 2-dose (10–39 days). For the 2-dose early cohort, mean Ct is about 1.54 Ct units higher than that of unvaccinated, corresponding to almost a threefold decrease in VL; However, this protection wanes rapidly as time elapses since vaccination, and Ct reaches a level similar to that of the unvaccinated by day 70. For the 3-dose (early) cohort, Ct is even higher than for the 2-dose (early) cohort, but once again rapid waning follows, and by day 70 Ct reaches the baseline level of the unvaccinated. Notably, Ct of the recovered cohort is similar to that of the 2-dose (early) and 3-dose (early) cohorts. Additional results for recovered individuals who were also vaccinated are presented in the Supplementary Information.

During the Omicron period (Fig. 1b), a recent 3rd-dose increases Ct among vaccinees, and is similar to infection-derived protection. Otherwise, the differences in Ct for the unvaccinated (adjusted Ct 25.9), 2-dose (adjusted Ct 25.7) and late 3-dose groups (adjusted Ct 25.8) are negligible (Fig. 1b). In general, the effect of immune status for Omicron is less pronounced than for Delta even upon recent receipt of the 3rd vaccine dose or a previous infection, as manifested by reduced Ct-values gaps between these groups and the unvaccinated. The relative difference between the recently vaccinated (3-dose, 10–39 days) and the unvaccinated is smaller in Omicron (0.97, 95% CI 0.78–1.16) compared to Delta (1.92, 95% CI 1.71–2.13). Similarly, the relative difference between recovered and unvaccinated is 1.69 (95% CI 1.49–1.88) in Delta, while in Omicron it is reduced to 0.78 (95% CI, 0.68–0.88). These gaps are reduced by a two-fold in Omicron, possibly due to host immune waning and viral evasion^9^ (see also Supplementary Table 2).

**Fig. 2:**
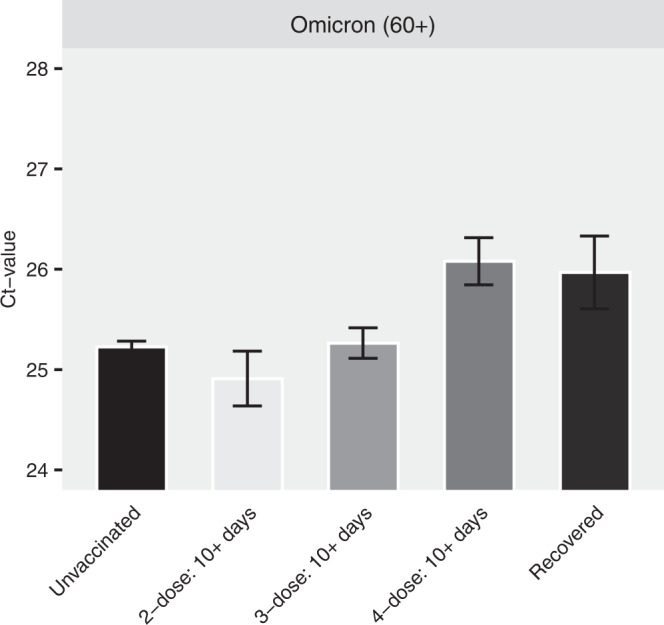
Ct values of the gene N for older adults during Omicron (*n* = 25,925 independent samples). Adjusted Ct values of different vaccination statuses for the Omicron variant in individuals of age >60. Means were obtained from the weighted sum of age, sex and calendar time, and the reference group (see Supplementary Table 2). Error bars represent 95% CI’s around the means, obtained by using the estimated distribution of all labs together (see Methods).

Since the 4-dose jab was administered mainly to older adults (60+, 90.9% of all 4-dose given in Israel), we conducted a separate analysis for this age group, pooling the 2- and 3-dose subgroups together. The results, presented in Fig. 2, reveal that shortly after receiving the 4th dose, Ct of the vaccinated individuals (ages > 60) reaches levels similar to those of recovered individuals, and significantly higher than those of the unvaccinated, 2- and 3- doses, from the same age group, indicating at least a short-term vaccine effectiveness of the vaccine in reducing Ct level (see also Supplementary Table 2). 4th dose effectiveness during the first month has also been demonstrated for confirmed infection and severe illness^10^.

**Fig. 3:**
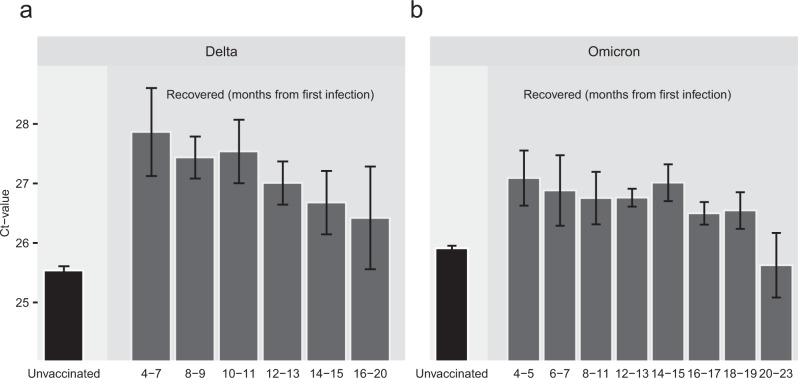
Ct values of the gene N for recovered, unvaccinated, individuals partitioned by number of months after recovery, for the Delta (a, *n* = 48,513 independent samples) and Omicron (*b*, n = 53,565 independent samples) variants. Means were obtained from the weighted sum of age, sex and calendar time, and the reference group (see Supplementary Table 4). Error bars represent 95% CI’s around the means, obtained by using the estimated distribution of all four labs together (see Methods). Individuals that were vaccinated between infection episodes were excluded from the recovered cohort.

Goldberg et al^11^. have shown that natural infection protection wanes over time, with a rate that is slower than that of vaccine-derived protection. Here we demonstrate that waning of natural infection protection pertains also to Ct, for both Delta and Omicron, likely indicating that vaccine-induced protection wanes with time. Figure 3 shows a clear and consistent waning trend for the recovered in Delta (Fig. 3a), while for the Omicron variant it is less clear (Fig. 3b). In contrast to the rapid waning observed for vaccinated individuals (Figs. 1 and 2), Ct of the previously infected in both variants demonstrates that VL levels remain low well beyond 18 months, and does not reach the baseline level of the unvaccinated even after extended periods of time (>18 months, see also Supplementary Table 4), and for individuals originally infected with the Wuhan or Alpha strains (Fig. 3).

## Discussion

While most studies concerning infectiousness of SARS-CoV-2 correlate Ct-values from qRT-PCR with pathogen burden as a surrogate for infectiousness potential^3,4,12–14^, other factors such as cell adhesion and cell entrance may play an additional role in explaining inter-strain virulence and infectiousness^15,16^. Infectivity is an intricate process comprised of numerous pathogen- and host-related factors, which are difficult to model in both humans and animals. Thus, Ct-values are still a commonly used proxy for infectiousness^4,7^. Nonetheless, some precaution should be taken interpreting Ct data in light of the above. While PCR efficiency is probably comparable for the Delta and Omicron variants, we cautiously refrain from comparing Ct values between variants and focus on within-variant comparisons. Other potential limitations of Ct data are lab-specific standards^17^ and temporal biases due to changing policies and health-seeking behaviors. To accommodate for these limitations, we used regressions, an approach that allows for accounting/adjusting for multiple factors. Particularly, temporal biases were addressed by the inclusion of calendric date as an explanatory variable in the regressions (see Supplementary Tables 2–5) (following^11^). In addition, we conducted additional sensitivity analyses which include separate single lab regressions (Supplementary Fig. 4), accounted for temporal biases using reproduction number (R) instead of calendar time (Supplementary Table 5, Analysis 1), and narrowed the period for Delta as well as restricting age ranges for both variants (Supplementary Table 5, Analysis 2). Finally, we also tested the robustness of the results by examining the patterns also for the gene E (Supplementary Fig. 3 and Supplementary Table 3).

This study indicates that overall the presumed vaccination-related immunity to SARS-CoV-2 has only a negligible long term (>70-days) effect on Ct value, a common surrogate for VL and infectiousness. The combination of vaccine waning and vaccine evasion are most likely the drivers of this finding. In lieu of several prominent publications describing vaccine effectiveness in prevention morbidity and hospitalization for Omicron^18–20^, this study mandates reevaluating the role of current vaccination campaigns in harnessing the potential infectivity of COVID-19 at a time scale >2 months. Consequently, different aspects of immunization benefits such as prevention and reduction of transmission (including duration of protection), severe disease, and mortality should be considered in planning booster vaccination campaigns. Decision makers should balance (i) judicious use of vaccine resources (ii) decreasing disease burden especially in high-risk populations (iii) false reassurance and promiscuous behavior due to the short-lived sterilizing immunity, which may deem vaccine campaigns as counterproductive epidemiologic restriction measure without proper communication with the public. Further studies should assess the differential benefits of SARS-CoV-2 vaccines in alleviating disease vs. preventing pathogen spread. Should the lack of sterilizing immunity prove consistent, it may have major ramifications on global pandemic preparedness, vaccination rollout and medical inequity. The demonstrated short-lived immunity and rapid waning on one hand, combined with the limited impact on population on the other, may focus the need for boosters for high-risk groups only, with immediate impact on vaccination campaigns and public health measures upon disease resurgence.

## Methods

### Ethics

The study was approved by an Institutional Review Board (IRB) of the Sheba Medical Center. Helsinki approval number: SMC-8228-21. Individuals included at this study were tested charge-free as part of the Israeli testing and surveillance program. Patient consent was waived, as this is a retrospective analysis using data which was collected as part of the national testing and surveillance program. The investigators did not have access to de-anonymized information.

### Dataset construction

We used a nationwide database of Ct values from positive cases. Samples were collected between June 15, 2021 and Jan 29, 2022. SAS and Python were used to retrieve the data, and R (version 4.0.3) and R Studio (version 1.4.1103) were used for analysis. The dataset contained over four million records of positive PCR tests with Ct-values. Records contained Ct measurements for the genes N, E, Orf1ab or S genes. Results are presented for N and E, and four different laboratories, two of which are major Israeli Health Maintenance Organization labs, together representing ~40% of the Israeli population, and the other two are labs commissioned to perform tests for the Israeli MoH. Ct values < 10 or >40 units were removed from the dataset, since such values are likely the result of reading errors. A negligible number of such samples were identified and removed from the four labs, N & E final dataset (9 records).

Multiple Ct measurements for the same individual and gene may belong to the same or different infection event. Sequences of Ct measurements within a single 90 days interval were defined as belonging to the same infection events. For each such sequence, only the first (earliest) Ct value was taken. Multiple infection events for a single individual were included if the time difference between the last measurement of the first sequence and the first measurement of the second sequence was at least 90 days. For the second infection, the patient’s status was defined as ‘Recovered’.

PCR and Ct values from over 460,000 were initially collected. Using encrypted identity numbers, we merged Ct data with demographic information and vaccination data, to determine the patient’s age, sex, and vaccination status. The merged data contained both Ct date and PCR sampling date. For the Ct measurements included in this study, the number of days between these two dates was at most a single day. Since PCR date is the actual sampling date, these dates were used for analyses. Overall, our analyses, performed on samples from four labs, and for the vaccination statuses detailed below, contained 327,659 individuals, 315,111 Ct measurements for the gene N, and 228,125 measurements for the gene E.

Vaccination statuses were determined for each patient and infection event, based on the PCR lab date. Individuals who had the infection between the first and the second dose were excluded from the analysis.

The following definitions were used to group individuals (Figs. 1 and 2):

Unvaccinated: Up to the first dose.

2-dose 10–39: From 10 days after the second dose up to 39 days after the second dose.

2-dose 40–69: From 40 days after the second dose up to 69 days after the second dose.

2-dose 70+: From 70 days onwards after the second dose, up to the third dose

3-dose 10–39: From 10 days after the third dose up to 39 days after the third dose.

3-dose 40–69: From 40 days after the third dose up to 69 days after the third dose.

3-dose 70+: From 70 days onwards after the third dose, up to the fourth dose

4-dose 10+: From 10 days onwards after the fourth dose.

Recovered: Individuals who had a previous infection event with a positive PCR test at least 90 days prior to the current infection, and who have not received a vaccination dose between the two events.

Recovered+vaccine: Individuals who had a previous infection event with a positive PCR test at least 90 days prior to the current infection, who received a single dose after the first infection event and no later than 10 days prior to the second infection event. Results for this group are presented only in Supplementary Information.

We divided the follow-up study into two separate periods, each dominated by a different variant:

Delta time period: June 15–Dec 1 2021 (>90% of the cases identified as Delta, see ref. 21).

Omicron time period: Dec 28 2021–Jan 29 2022 (>90% of the cases identified as Omicron, see ref. 21).

These periods are also in accordance with the first documented case of Omicron infection in Israel (see Supplementary Fig. 1).

To account for temporal effects in our regression analyses, we partitioned the Delta and Omicron time periods into 7-day time intervals (bins), using PCR dates to classify Ct measurements. Age groups were defined as 0–11, 12–15, 16–39, 40–59, and 60 or older. Due to national policy, individuals of age 0–11 were not vaccinated until relatively late, and were thus excluded from the main analysis. Nonetheless, ages 5–11 were included in parts of the sensitivity analysis presented in Supplementary Note 1 (see Supplementary Table 5).

For the analysis of waning in the ‘Recovered’ (Fig. 3), we partitioned the time elapsed between the first and second infections into 60-days intervals (2 months). Intervals for which the number of samples was <50 were merged with the adjacent interval.

### Statistical analysis

The main tools in assessing the effect of different factors on Ct-values are the linear and quantile regressions. On examination of the different cohorts, we used cohort, age category, sex, and categorized calendar date as explanatory variables. Daily Ct values may have been sampled from infectees at different stages of the infection (i.e., time from infection). We thus also examined the median and a lower quartile of Ct values, controlling for age-of-infection variability (see Supplementary Tables 2 and 3). To compute error bars in Figs. 1–3, as well as Supplementary Figs. 2–4, we used the estimated cohort coefficients, while setting all other coefficients to their mean values (as in predictor effect plots^22^). We then calculated the (0.025,0.975)-percentiles to obtain confidence intervals through the multivariate normal distribution.

### Reporting summary

Further information on research design is available in the Nature Research Reporting Summary linked to this article.

## Supplementary information

Supplementary Information

Reporting Summary

## Data Availability

The individual-level data cannot be publicly shared due to Israel Ministry of Health’s regulations. Requests for remote access to de-identified data should be referred to naamako@hit.ac.il, and will be assisted within 4 weeks pending IRB approval. Source data are provided with this paper.

## Source data

Source Data

## Code availability

Code for producing main figures and analyses is available at https://github.com/yonatan123/Viral-load. Full code version will be shared upon request.

## References

[1] Dagan N (2021). BNT162b2 mRNA Covid-19 vaccine in a nationwide mass vaccination setting. N. Eng. J. Med..

[2] Bar-On YM (2021). Protection of BNT162b2 vaccine booster against Covid-19 in Israel. N. Eng. J. Med..

[3] Marks M (2021). Transmission of COVID-19 in 282 clusters in Catalonia, Spain: a cohort study. Lancet Infect. Dis..

[4] Lyngse, F. P. et al. Association between SARS-CoV-2 transmissibility, viral load, and age in Danish households. *medRxiv*10.1101/2021.02.28.21252608 (2021).

[5] Israel Ministry of Health. Coronavirus in Israel - general update. https://datadashboard.health.gov.il/COVID-19/general (2022).

[6] Levine-Tiefenbrun M (2021). Initial report of decreased SARS-CoV-2 viral load after inoculation with the BNT162b2 vaccine. Nat. Med..

[7] Levine-Tiefenbrun M (2021). Viral loads of Delta-variant SARS-CoV-2 breakthrough infections after vaccination and booster with BNT162b2. Nat. Med..

[8] Levine-Tiefenbrun M (2022). Waning of SARS-CoV-2 booster viral-load reduction effectiveness. Nat. Comm..

[9] Levin EG (2021). Waning immune humoral response to BNT162b2 Covid-19 vaccine over 6 months. N. Eng. J. Med..

[10] Bar-On YM (2022). Protection by a fourth dose of BNT162b2 against Omicron in Israel. N. Eng. J. Med..

[11] Goldberg Y (2021). Protection and waning of natural and hybrid immunity to SARS-CoV-2. N. Eng. J. Med..

[12] Kawasuji H (2020). Transmissibility of COVID-19 depends on the viral load around onset in adult and symptomatic patients. PloS ONE.

[13] Al Bayat S (2021). Can the cycle threshold (Ct) value of RT-PCR test for SARS CoV2 predict infectivity among close contacts?. J. Infect. Public Health.

[14] Trunfio, M. et al. Determinants of SARS-CoV-2 contagiousness in household contacts of symptomatic adult index cases. *Front. Microbiol*. **13** (2022). 10.3389/fmicb.2022.829393 PMC9010948 35432272

[15] Hay, J. A. et al. Viral dynamics and duration of PCR positivity of the SARS-CoV-2 Omicron variant. *medRxiv*10.1101/2022.01.13.22269257 (2022).

[16] Puhach, O. et al. Infectious viral load in unvaccinated and vaccinated individuals infected with ancestral, Delta or Omicron SARS-CoV-2. *Nat. Med.* **28**, 1491–1500 (2022). 10.1038/s41591-022-01816-0 35395151

[17] Evans D (2022). The dangers of using Cq to quantify nucleic acid in biological samples: a lesson from COVID-19. Clin. Chem..

[18] Collie S, Moultrie H, Bekker LG, Gray G (2021). Effectiveness of BNT162b2 vaccine against Omicron variant in South Africa. N. Eng. J. Med..

[19] UK Health Security Agency. COVID-19 vaccine surveillance report – week 6. publishing.service.gov.uk (2022).

[20] Thompson MG (2022). Effectiveness of a third dose of mRNA vaccines against COVID-19-associated emergency department and urgent care encounters and hospitalizations among adults during periods of Delta and Omicron variant predominance - VISION Network, 10 States, August 2021-January 2022. MMWR Morb. Mortal. Wkly. Rep..

[21] Our World in Data. https://ourworldindata.org/ (2022).

[22] Fox J, Weisberg S (2018). Visualizing fit and lack of fit in complex regression models with predictor effect plots and partial residuals. J. Stat. Softw..

