# Supplementary material for: Viral load dynamics of SARS-CoV-2 Delta and Omicron variants following multiple vaccine doses and previous infection
Source: Nat Commun. 2022 Nov 7;13:6706. doi: 10.1038/s41467-022-33096-0 (PMC9640564; doi:10.1038/s41467-022-33096-0)
Supplement: Supplementary file 1 — Supplementary Information [file 41467_2022_33096_MOESM1_ESM.pdf]

Viral load dynamics of SARS-CoV-2 Delta and Omicron variants following multiple vaccine doses and previous infection - Supplementary Information

Data description: sample counts, means and medians for N-gene Ct values

		Delta			Omicron		
Lab	Cohort	N	Mean(s.d)	Median	N	Mean(s.d)	Median
Lab1	Unvaccinated	13100	25.4 (5.04)	24.72	19928	26.07 (4.74)	25.65
	2-dose: 10-39	141	27.57 (4.87)	26.91	170	25.72 (4.45)	25.25
	2-dose: 40-69	223	26.37 (4.77)	25.87	142	26.74 (4.84)	26.81
	2-dose: 70+	15247	25.36 (5.01)	24.48	11230	26.03 (4.78)	25.59
	3-dose: 10-39	657	28.19 (4.59)	28.39	1171	27.05 (4.6)	26.69
	3-dose: 40-69	252	26.8 (4.6)	26.52	696	26.54 (4.73)	26.11
	3-dose: 70+	113	27.08 (4.27)	26.76	44526	25.94 (4.74)	25.49
	4-dose				1551	25.72 (4.6)	25.48
	Recovered	638	27.66 (4.89)	27.74	4237	27.14 (4.55)	26.95
	Recovered+vaccine	82	27.55 (5.99)	28.11	2248	27.09 (4.64)	26.72
Lab2	Unvaccinated	8870	26.59 (5.64)	25.89	705	26.42 (4.18)	26.35
	2-dose: 10-39	57	27.08 (5.72)	25.95	1	26.34 ()	26.34
	2-dose: 40-69	105	27.64 (5.69)	26.45	4	26.63 (5.18)	27.67
	2-dose: 70+	6634	26.22 (5.51)	25.26	342	25.93 (4.52)	25.79
	3-dose: 10-39	340	27.91 (5.48)	27.25	6	29.09 (1.96)	29.13
	3-dose: 40-69	135	28.38 (5.82)	28	15	27.13 (4.22)	26
	3-dose: 70+	63	26.49 (5.66)	25.75	659	25.47 (4.48)	25.18
	4-dose				1	34.2 ()	34.2
	Recovered	789	28.12 (5.59)	27.76	253	26.56 (4.45)	26.34
	Recovered+vaccine	121	27.61 (5.83)	27.01	110	26.65 (4.01)	26.08
Lab3	Unvaccinated	13584	25.01 (4.61)	24.62	16153	25.87 (4.01)	25.79
	2-dose: 10-39	90	26.52 (4.18)	25.98	85	25.41 (3.8)	25.59
	2-dose: 40-69	144	25.91 (4.32)	25.45	87	25.69 (3.58)	25.22
	2-dose: 70+	15060	24.74 (4.54)	24.19	10189	25.64 (3.98)	25.5
	3-dose: 10-39	553	26.56 (4.36)	26.34	596	26.93 (3.84)	27.04
	3-dose: 40-69	154	26.65 (4.37)	26.36	641	26.19 (3.97)	26.08
	3-dose: 70+	68	26.23 (4.69)	26.18	26661	25.78 (4.03)	25.64
	4-dose				724	26.33 (4.24)	26.38
	Recovered	467	26.55 (4.61)	26.61	2686	26.29 (4)	26.25
	Recovered+vaccine	89	25.08 (4.67)	24.55	1978	26.45 (3.89)	26.39
Lab4	Unvaccinated	11459	25.52 (4.62)	25.03	8796	25.64 (4.4)	25.97
	2-dose: 10-39	79	26.57 (4.68)	26.03	59	25.62 (4.8)	25.03
	2-dose: 40-69	168	25.8 (4.5)	25.03	44	27.26 (4.68)	27.5
	2-dose: 70+	11623	25.24 (4.52)	25.03	4924	25.46 (4.41)	25.03
	3-dose: 10-39	716	26.8 (4.42)	27.03	351	26.12 (4.54)	26.03
	3-dose: 40-69	353	26.49 (4.58)	26.03	365	26.21 (4.44)	26.03
	3-dose: 70+	145	27.08 (4.58)	27.03	19869	25.37 (4.45)	25.03
	4-dose				222	24.82 (4.32)	25.03
	Recovered	644	26.79 (4.16)	27.03	1944	26.56 (4.27)	26.97
	Recovered+vaccine	87	26.34 (4.58)	26.03	1159	26.38 (4.44)	26.03

Supplementary Table 1. Number of samples, mean (s.d.) and median of all N-gene Ct values, grouped by laboratory, cohort and gene, for Delta and Omicron time periods. Only ages 12 and above were included in this table.

Regression results for the gene N

Variant	Delta (See Fig. 1a, main text)		Omicron (see Fig. 1b, main text)		Omicron (60+) (see Fig. 2, main text)
Model	Linear model	Quantiles 0.5 (0.2)	Linear model	Quantiles 0.5 (0.2)	Linear model
**Vaccination status**					
Unvaccinated	ref.	ref.	ref.	ref.	ref.
2-dose: 10-39	1.54 (1.03,2.04) *	1.59* (1.58*)			
2-dose: 40-69	0.86 (0.48,1.25) *	0.85* (0.94*)			
2-dose: 70-	-0.17 (-0.24, -0.1) *	-0.26* (0)			
2-dose (All)			-0.15 (-0.22, -0.08) *	-0.18* (-0.21*)	-0.32 (-0.58, -0.05) *
3-dose: 10-39	1.92 (1.71,2.13) *	2.3* (1.99*)	0.97 (0.78,1.16) *	1.15* (1.12*)	
3-dose: 40-69	0.97 (0.64,1.3) *	0.95* (1.24*)	0.41 (0.19,0.62) *	0.34* (0.43*)	
3-dose: 70-	0.34 (-0.18,0.86)	0.12 (0.91*)	-0.12 (-0.18, -0.07) *	-0.18* (-0.18*)	
3-dose (All)					0.04 (-0.1,0.18)
4-dose					0.85 (0.62,1.08) *
Recovered	1.69 (1.49,1.88) *	2.07* (1.63*)	0.78 (0.68,0.88) *	0.93* (0.85*)	0.74 (0.38,1.1) *
Recovered+vaccine	1.03 (0.53,1.52) *	1.31* (0.74*)	0.76 (0.64,0.89) *	0.77* (0.86*)	1.38 (0.92,1.84) *
**Age**					
12-15	0.01 (-0.08,0.11)	0.01 (-0.06)	-0.16 (-0.24, -0.09) *	-0.22* (-0.21*)	
16-39	ref.	ref.	ref.	ref.	
40-59	0.02 (-0.06,0.09)	0.03 (0)	-0.01 (-0.05,0.04)	0 (-0.06)	
60+	-0.21 (-0.32, -0.09) *	-0.16 (-0.15*)	-0.66 (-0.72, -0.59) *	-0.71* (-0.61*)	
**Sex**					
Female	ref.	ref.	ref.	ref.	ref.
Male	0.1 (0.04,0.16) *	0.19* (0.06*)	-0.16 (-0.2, -0.12) *	-0.13* (-0.18*)	-0.29 (-0.4, -0.18) *
**Lab**					
Lab1	ref.	ref.	ref.	ref.	ref.
Lab2	0.97 (0.88,1.07) *	0.91* (0.46*)	-0.63 (-0.83, -0.42) *	-0.61* (-0.09)	-0.86 (-1.51, -0.22) *
Lab3	-0.57 (-0.65, -0.49) *	-0.27* (-0.09*)	-0.3 (-0.35, -0.26) *	0 (0.33*)	0.13 (0.01,0.26) *
Lab4	-0.13 (-0.21, -0.04) *	0.4* (0.33*)	-0.75 (-0.81, -0.7) *	-0.49* (-0.5*)	-0.17 (-0.33, -0.02) *
**Calendar time**					
0	ref.	ref.	ref.	ref.	ref.
1	0.1 (-1.01,1.21)	-0.29 (0.34)	-0.18 (-0.26, -0.1) *	-0.46* (0.08)	-0.2 (-0.44,0.05)
2	0.38 (-0.71,1.47)	-0.73 (0.33)	-0.46 (-0.55, -0.38) *	-0.81* (-0.21*)	-0.62 (-0.87, -0.38) *
3	0.25 (-0.84,1.34)	-0.64 (0.15)	-0.64 (-0.72, -0.55) *	-0.81* (-0.28*)	-0.85 (-1.1, -0.61) *
4	-0.18 (-1.24,0.88)	-0.99 (0.03)	-0.96 (-1.06, -0.87) *	-1.08* (-0.61*)	-1.35 (-1.62, -1.08) *
5	-0.28 (-1.32,0.76)	-1.18 (0.04)			
6	-0.08 (-1.11,0.96)	-0.99 (0.04)			
7	0.47 (-0.56,1.5)	-0.33 (0.68)			
8	-0.06 (-1.09,0.97)	-0.86 (0.31)			
9	-0.06 (-1.09,0.97)	-0.89 (0.44)			
10	-0.35 (-1.38,0.68)	-1.07 (0.23)			
11	-0.27 (-1.29,0.76)	-1.07 (0.25)			
12	-0.32 (-1.35,0.71)	-1.01 (0.25)			
13	-0.2 (-1.23,0.83)	-0.87 (0.31)			

14	0.07 (-0.96,1.1)	-0.73 (0.72)			
15	0.3 (-0.74,1.33)	-0.21 (0.79)			
16	0.38 (-0.66,1.42)	-0.09 (1.03)			
17	0.59 (-0.46,1.64)	-0.02 (1.06)			
18	0.59 (-0.48,1.65)	0.28 (0.99)			
19	1.5 (0.42,2.58) *	1.28 (1.95)			
20	0.94 (-0.14,2.02)	0.21 (1.25)			
21	0.88 (-0.23,2)	0.29 (1.25)			
22	0.86 (-0.25,1.96)	0.34 (1.27)			
23	1.01 (-0.09,2.11)	1.04 (1.25)			
24	1.7 (0.3,3.09) *	2.01 (2.09)			

Supplementary Table 2. Extended regression results for N-gene Ct values. Linear and quantile regression results on Ct-value, estimated separately for Delta and Omicron time periods. Linear regressions are the primary analyses discussed and presented in the main text; quantile regressions were conducted as secondary analyses. The revealed trends are highly similar between the linear and quantile analyses. 95% CI are given in parentheses. For quantile regression, median and 0.2 quartile (in parentheses) are provided. (*) denote 0.05 significance. Calendar time was partitioned to 1-week intervals, starting from June 15, 2021 for the Delta, and from Dec 28, 2021 for the Omicron time periods. Only ages 12 and above were included.

Regression results for the gene E

Variant	Delta		Omicron		Omicron (60+)
Model	Linear model	Quantiles 0.5 (0.2)	Linear model	Quantiles 0.5 (0.2)	Linear model
**Vaccination status**					
Unvaccinated	ref.	ref.	ref.	ref.	ref.
2-dose: 10-39	1.37 (0.81,1.93) *	1.54* (1.68*)			
2-dose: 40-69	0.72 (0.28,1.15) *	0.69 (0.84*)			
2-dose: 70-	-0.2 (-0.28, -0.12) *	-0.32* (-0.05)			
2-dose (All)			-0.15 (-0.24, -0.07) *	-0.11* (-0.12*)	-0.41 (-0.74, -0.08) *
3-dose: 10-39	1.99 (1.76,2.23) *	2.43* (2.31*)	0.91 (0.69,1.14) *	1.06* (0.81*)	
3-dose: 40-69	0.96 (0.59,1.32) *	0.98* (1.18*)	0.48 (0.21,0.74) *	0.71* (0.39)	
3-dose: 70+	0.15 (-0.4,0.69)	0.18 (0.6*)	-0.11 (-0.17, -0.05) *	-0.11* (-0.09*)	
3-dose (All)					0.02 (-0.15,0.19)
4-dose					0.99 (0.72,1.26) *
Recovered	1.7 (1.48,1.91) *	2.01* (1.86*)	0.81 (0.7,0.93) *	0.95* (0.98*)	0.89 (0.47,1.31) *
Recovered+vaccine	1.31 (0.76,1.87) *	1.77* (0.71*)	0.78 (0.63,0.93) *	0.88* (0.97*)	1.25 (0.68,1.82) *
**Age**					
12-15	0.04 (-0.08,0.15)	0.05 (-0.02)	0 (-0.09,0.1)	0 .01(-0.03)	
16-39	ref.	ref.	ref.	ref.	
40-59	0 (-0.09,0.09)	0.02 (0)	-0.07 (-0.12, -0.01) *	-0.06 (-0.06*)	
60+	-0.24 (-0.38, -0.11) *	-0.17 (-0.21*)	-0.69 (-0.76, -0.61) *	-0.81* (-0.61*)	
**Sex**					
Female	ref.	ref.	ref.	ref.	ref.
Male	0.03 (-0.04,0.1)	0.14* (0.01)	-0.26 (-0.31, -0.21) *	-0.18* (-0.09*)	-0.38 (-0.51, -0.25) *
**Lab**					
Lab1	ref.	ref.	ref.	ref.	ref.
Lab2	3.61 (3.51,3.71) *	3.59* (3.21*)	1 (0.78,1.21) *	1.15* (1.57*)	0.94 (0.27,1.61) *
Lab3	6.29 (6.15,6.43) *	8.2* (5.5*)	5.25 (5.14,5.37) *	5.85* (7.34*)	5.6 (5.26,5.94) *
Lab4	1.77 (1.68,1.86) *	2.29* (2.3*)	1.05 (0.99,1.11) *	1.26* (1.48*)	1.65 (1.48,1.81) *
**Calendar time**					
0	ref.	ref.	ref.	ref.	ref.
1	2.23 (0.95,3.5) *	2.23* (2.25)	-1.1 (-1.2, -0.99) *	-1.1* (-1.01*)	-0.99 (-1.29, -0.69) *
2	2.03 (0.78,3.28) *	1.24 (2.11)	-1.71 (-1.81, -1.61) *	-1.86* (-1.55*)	-1.67 (-1.96, -1.37) *
3	2.42 (1.18,3.65) *	1.56* (2.2)	-1.51 (-1.61, -1.41) *	-1.55* (-1.16*)	-1.53 (-1.84, -1.23) *
4	1.96 (0.76,3.16) *	1.37 (2.24*)	-0.85 (-0.97, -0.74) *	-0.92* (-0.45*)	-0.96 (-1.29, -0.64) *
5	1.59 (0.41,2.77) *	0.76 (1.95)			
6	2.03 (0.86,3.2) *	1.3 (2.35*)			
7	2.54 (1.37,3.7) *	1.95* (2.8*)			
8	2.27 (1.1,3.43) *	1.71* (2.75*)			
9	2.07 (0.91,3.23) *	1.41* (2.54*)			
10	2.2 (1.03,3.36) *	1.71* (2.69*)			
11	2 (0.84,3.16) *	1.28 (2.12)			
12	1.95 (0.78,3.11) *	1.28 (2.04)			
13	2.01 (0.85,3.17) *	1.29 (2.21*)			
14	1.97 (0.81,3.14) *	1.22 (2.35*)			
15	2.45 (1.28,3.62) *	1.96* (2.75*)			
16	2.43 (1.25,3.6) *	1.99* (2.74*)			
17	2.58 (1.4,3.77) *	1.96* (2.77*)			

18	2.48 (1.28,3.68) *	1.95* (2.74*)			
19	3.27 (2.05,4.49) *	2.96* (3.74*)			
20	2.93 (1.7,4.15) *	2.23* (3.35*)			
21	2.83 (1.58,4.08) *	2.42* (2.89*)			
22	3.02 (1.78,4.25) *	2.58* (3.47*)			
23	2.63 (1.41,3.86) *	2.43* (2.74*)			
24	3.51 (1.96,5.06) *	4.78* (4.16*)			

Supplementary Table 3. Extended regression results for E-gene Ct-values. Linear and quantile regression results on Ct-value, estimated separately for Delta and Omicron time periods. 95% CI are given in parentheses. For quantile regression, median and 0.2 quartile (in parentheses) are provided. (*) denote 0.05 significance. Calendar time was partitioned to 1-week intervals, starting from June 15, 2021 for the Delta, and from Dec 28, 2021 for the Omicron time periods. Only ages 12 and above were included.

Regression results for the gene N & Recovered individuals

Variant	Delta (See Fig. 3a, main text)	Omicron (see Fig. 3b, main text)
**Vaccination status**		
Unvaccinated	ref.	ref.
Recovered: months 4-5		1.18 (0.72,1.64) *
Recovered: months 6-7		0.97 (0.38,1.56) *
Recovered: months 4-7	2.33 (1.59,3.07) *	
Recovered: months 8-9	1.9 (1.55,2.25) *	
Recovered: months 10-11	2 (1.47,2.53) *	
Recovered: months 8-11		0.84 (0.4,1.29) *
Recovered: months 12-13	1.47 (1.12,1.83) *	0.85 (0.71,0.99) *
Recovered: months 14-15	1.14 (0.61,1.67) *	1.1 (0.8,1.41) *
Recovered: months 16-17		0.59 (0.4,0.77) *
Recovered: months 16-20	0.89 (0.03,1.75) *	
Recovered: months 18-19		0.63 (0.33,0.94) *
Recovered: months 20-23		-0.29 (-0.83,0.25)
**Age**		
12-15	-0.02 (-0.12,0.09)	-0.02 (-0.12,0.09)
16-39	ref.	ref.
40-59	-0.04 (-0.16,0.08)	-0.13 (-0.22, -0.04) *
60+	-0.16 (-0.35,0.03)	-0.8 (-0.93, -0.68) *
**Sex**		
Female	ref.	ref.
Male	0.17 (0.08,0.26) *	-0.15 (-0.22, -0.07) *
**Lab**		
Lab1	ref.	ref.
Lab2	1.1 (0.97,1.23) *	-0.4 (-0.72, -0.08) *
Lab3	-0.49 (-0.61, -0.37) *	-0.4 (-0.48, -0.31) *
Lab4	-0.08 (-0.21,0.04)	-0.68 (-0.79, -0.58) *
**Calendar time**		
0	ref.	ref.
1	0.08 (-0.89,1.06)	-0.15 (-0.31,0.01) *
2	0.21 (-0.75,1.18)	-0.42 (-0.57, -0.26) *
3	-0.19 (-1.18,0.8)	-0.58 (-0.74, -0.42) *
4	-0.63 (-1.5,0.25)	-0.92 (-1.1, -0.74) *
5	-0.68 (-1.51,0.16)	
6	-0.01 (-0.83,0.8)	
7	0.14 (-0.66,0.94)	
8	-0.51 (-1.32,0.29)	
9	-0.35 (-1.15,0.44)	
10	-0.43 (-1.22,0.37)	
11	-0.41 (-1.2,0.38)	
12	-0.3 (-1.1,0.49)	
13	-0.24 (-1.03,0.56)	
14	0.1 (-0.71,0.9)	
15	0.24 (-0.57,1.05)	
16	0.25 (-0.57,1.08)	
17	0.48 (-0.37,1.33)	

18	0.78 (-0.11,1.66)	
19	1.19 (0.3,2.09) *	
20	0.54 (-0.4,1.48)	
21	0.74 (-0.21,1.7)	
22	0.96 (0.02,1.91) *	
23	1.45 (0.43,2.46) *	

Supplementary Table 4. Linear regression analysis results for gene N Ct values on recovered patients. 95% CI are given in parentheses. (*) denote 0.05 significance. Calendar time was partitioned to 1-week intervals, starting from June 18, 2021 for the Delta and from Dec 28, 2021 for the Omicron time periods. Only ages 12 and above were included.

Supporting Information Figures

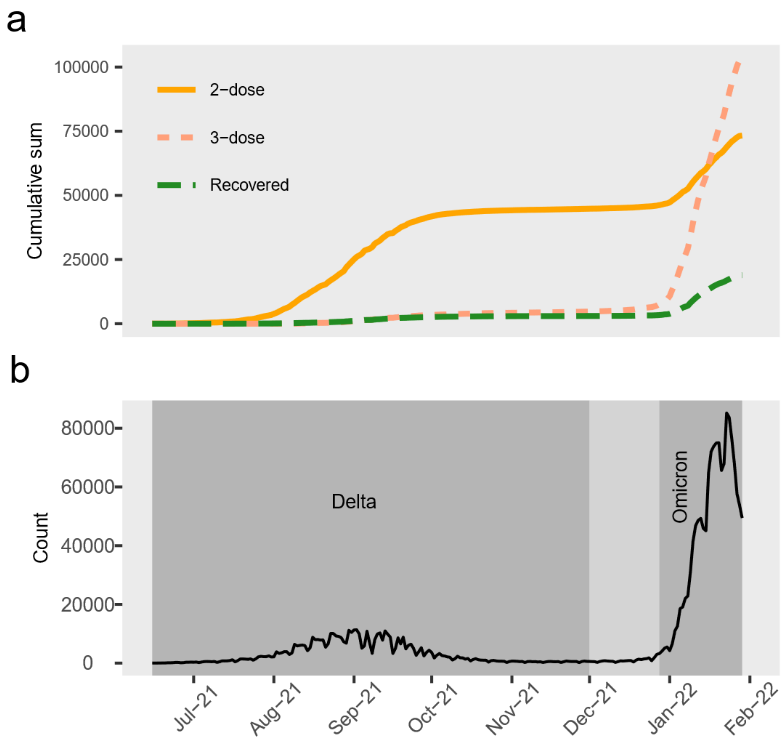

Supplementary Fig. 1. a: The cumulative number of N-gene Ct measurements by date, for 2-does, 3-dose, and recovered individuals, for a combined dataset of all four labs, from June 15, 2021until Jan 29, 2022. **b: Number of positive cases, as reported by the Israeli Ministry of Health**. Shaded areas highlight the Delta and Omicron time periods, as defined in the main text.

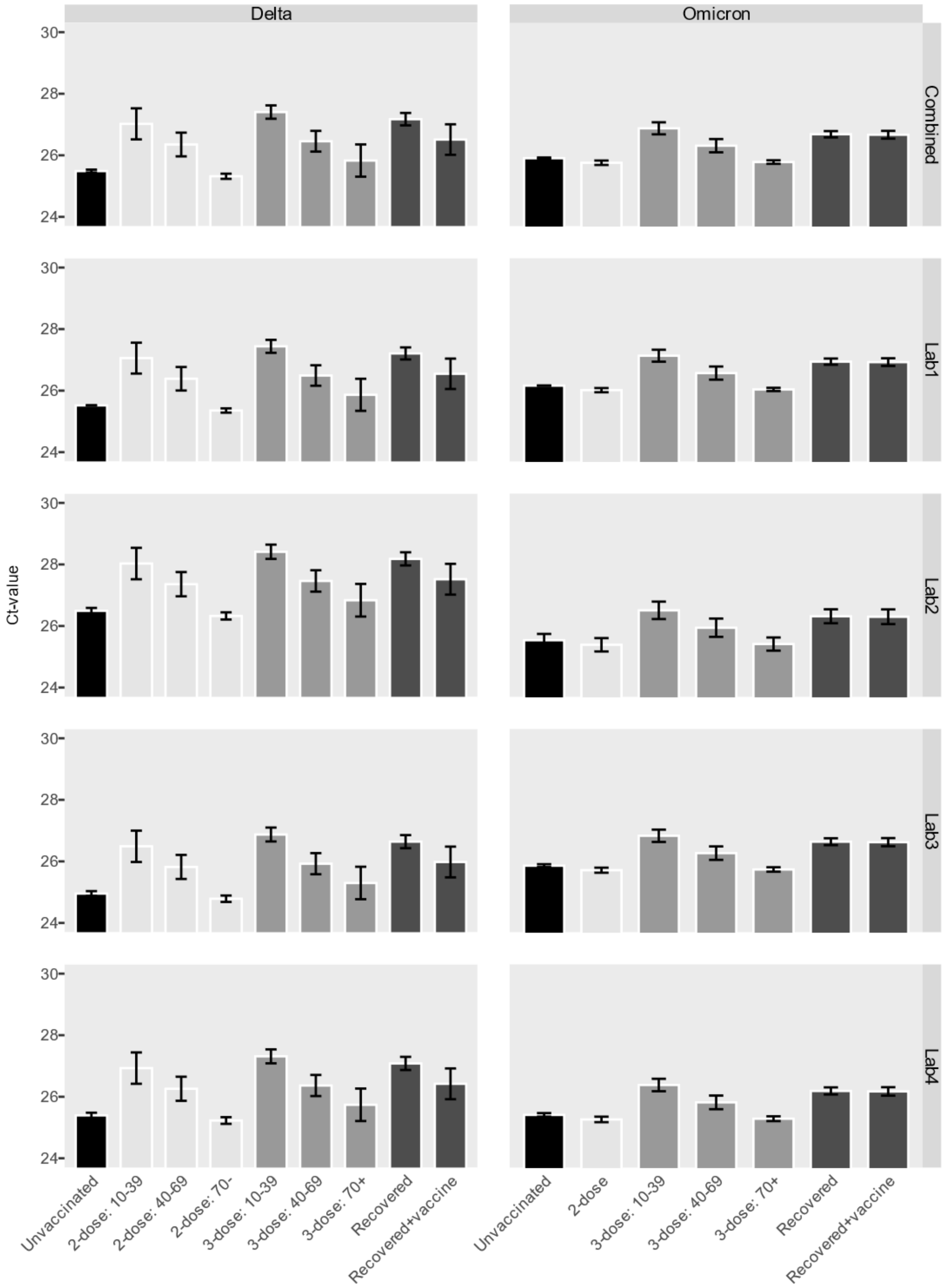

Supplementary Fig. 2. N-gene Ct values for different vaccination statuses, measured by four laboratories and combined, for the Delta (left panel, n=101,897 independent samples) and Omicron (right panel, n=181,634 independent samples) variants, using the multivariate regression coefficients (see Supplementary Table 2). Results are provided for each lab in a separate row, demonstrating similar patterns in all labs. Error bars represent 95% CI's around the means. Means were obtained from the weighted sum of age, sex and calendar time (using their frequency for each variant), together with the reference group (see Supplementary Table 2), and the corresponding cohort and lab for each column bar. CI's were obtained using the estimated distribution of each pair of cohort-lab coefficients. As to "Combined", CI's were obtained from the estimated distribution of all labs together with each of the cohorts' coefficients. Only ages 12 and above were included.

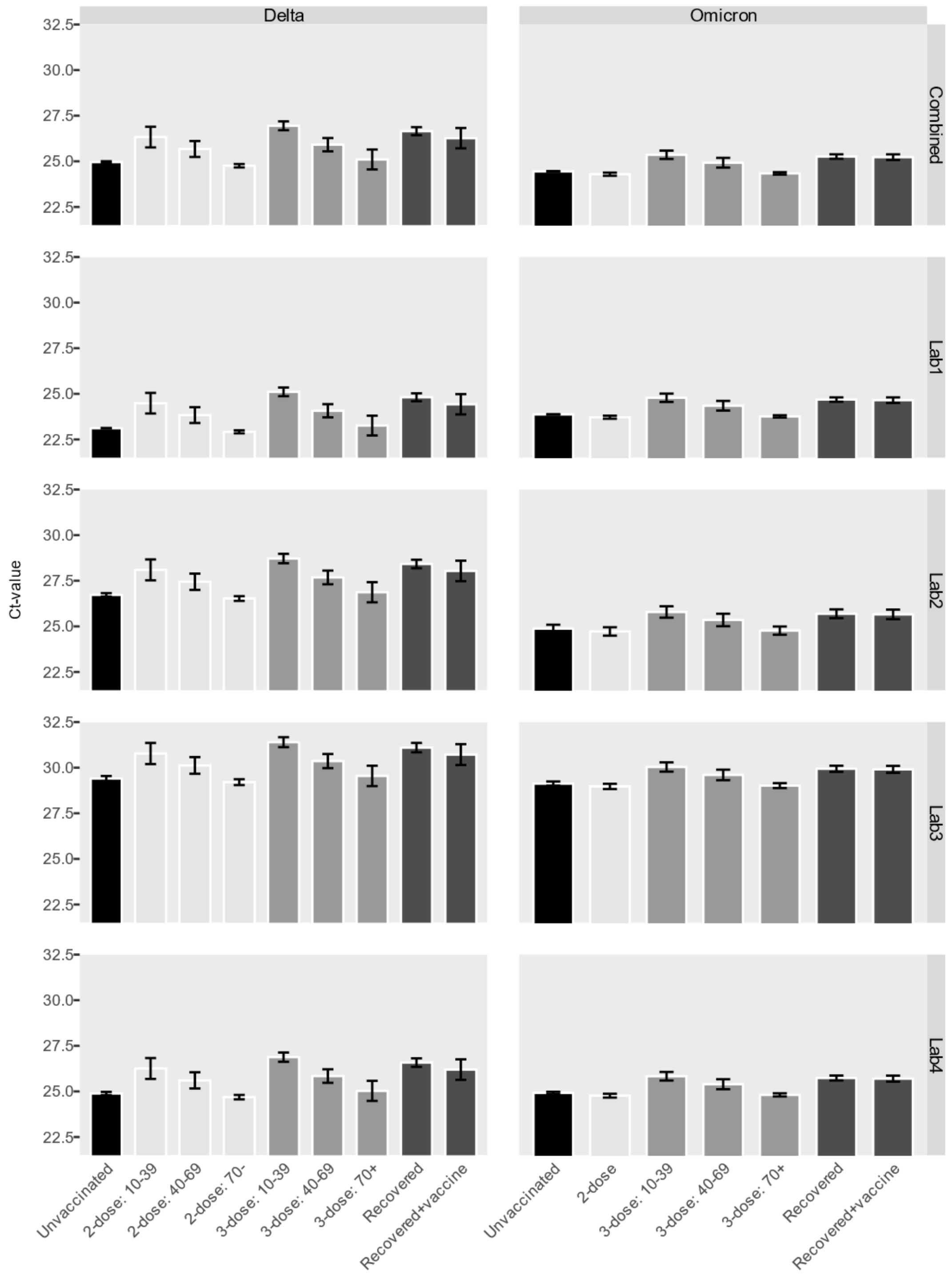

Supplementary Fig. 3. E-gene Ct values for different vaccination statuses, measured by four laboratories and combined, for the Delta (left panel, n=78,222 independent samples) and Omicron (right panel, n=128,777 independent samples), using the multivariate regression coefficients. Results are provided for each lab in a separate row, demonstrating similar patterns in all labs. Error bars represent 95% CI's around the means. Means were obtained from the weighted sum of age, sex and calendar time (using their frequency for each variant), together with the reference group (see Supplementary Table 3), and the corresponding cohort and lab for each column bar. CI's were obtained using the estimated distribution of each pair of cohort-lab coefficients. As to "Combined", CI's were obtained from the estimated distribution of all labs together with each of the cohorts' coefficients. Note that both E and N have similar patterns (Supplementary Figs. 2-3). Only ages 12 and above were included.

Supplementary Note 1: Sensitivity Analysis

Supplementary Fig. 4 presents the results of regression analyses performed separately on each lab, in order to examine each lab independently, thus accounting for lab variability. The consistency of the results supports our assumption that vaccination status affects Ct values in a similar manner across all labs, regardless of different lab procedures and measurement standards.

Supplementary Table 5 presents two parts of a sensitivity analysis. In Analysis 1, we performed linear regression with the same covariates as in Table 2, but replaced calendar time with the R variable, thus accounting for temporal effects in a different manner. We observe similar patterns in terms of the coefficients for Delta and Omicron, as shown in Table 5. In Analysis 2, we used a restricted follow-up study time for Delta (between Sep 07 and Oct 11, 2021), as well as certain ages for both variants to mitigate temporal and age effects (12-20 for Delta, 5-20 for Omicron; Note that for Omicron, ages 5-11 that were excluded from the main analyses are included here).

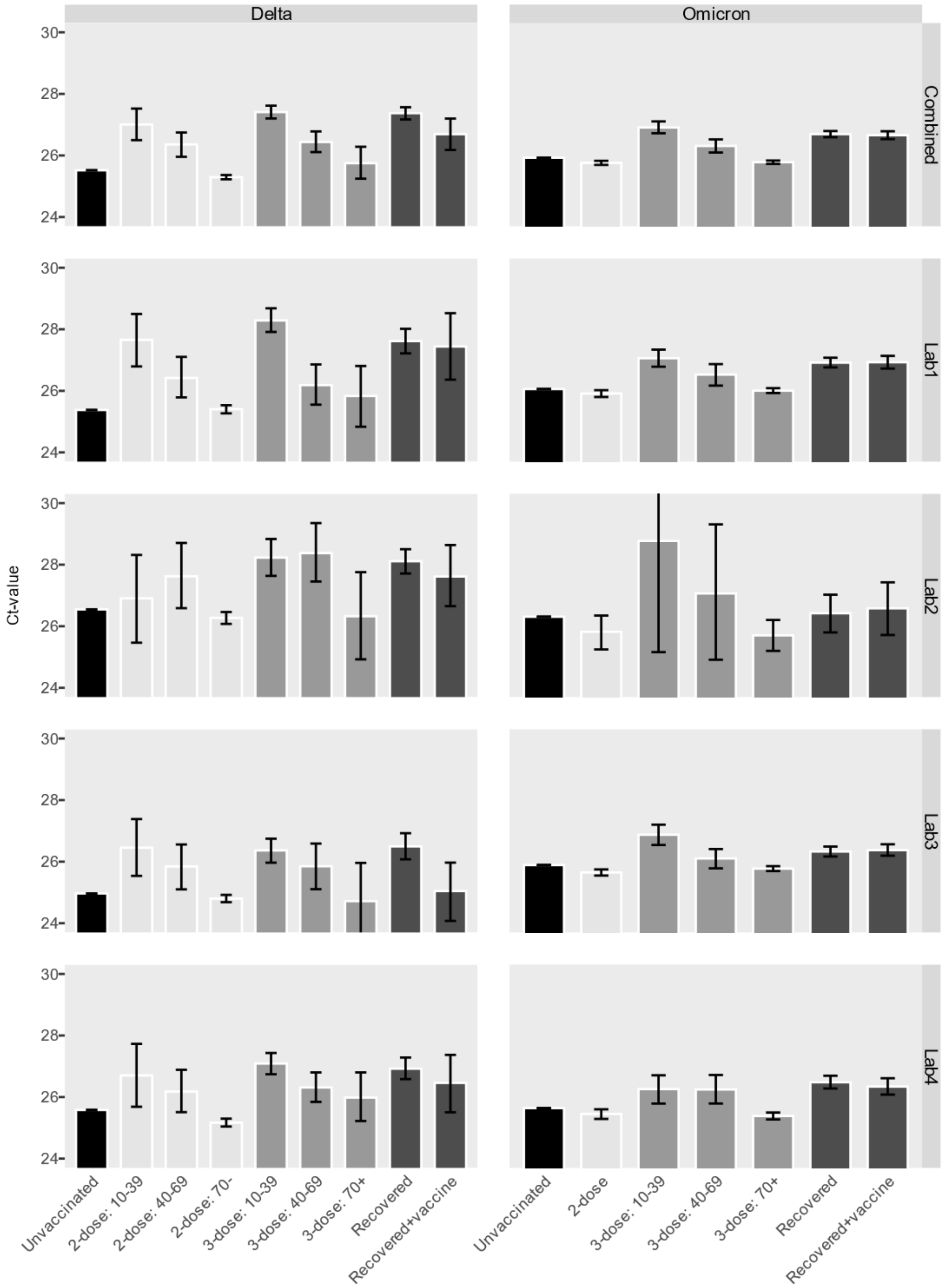

Supplementary Fig. 4. N-gene Ct values for different vaccination cohorts, for the Delta (left panel, n=101,897 independent samples) and Omicron (right panel, n=181,634 independent samples) variants, based on regression analyses performed separately for each lab (for number of samples within each lab, see Supplementary Table 1). Error bars represent 95% CI's around the means. Means were obtained from the weighted sum of age, sex and calendar time (using their frequency for each pair of lab-variant), together with the reference group (reference group taken from Supplementary Table 2), and the corresponding cohort for each column bar. As to 'Combined', means were obtained from the weighted sum of age, sex, calendar time and lab, together with the reference group and the corresponding cohort for each column bar. CI's were obtained using the estimated distribution of each of the cohort coefficients.

Sensitivity analysis table

	Analysis 1: with R		Analysis 2:	
	Delta	Omicron	Delta (dates: Sep 07-Oct 11 2021, ages: 12-20)	Omicron (ages: 5-20)
**Cohort**				
Unvaccinated	ref.	ref.	ref.	ref.
2-dose: 10-39	1.5 (0.99,2) *		1.1 (0.05,2.14) *	-0.36 (-0.53, -0.19) *
2-dose: 40-69	0.79 (0.41,1.17) *		0.87 (0.35,1.4) *	-0.39 (-0.85,0.08)
2-dose: 70-	-0.2 (-0.27, -0.13) *		-0.23 (-0.49,0.03)	-0.82 (-0.91, -0.73) *
2-dose (All)		-0.15 (-0.21, -0.08) *		
3-dose: 10-39	1.86 (1.65,2.07) *	0.97 (0.77,1.16) *		0.34 (0.08,0.59) *
3-dose: 40-69	1.45 (1.12,1.78) *	0.4 (0.18,0.61) *		-0.21 (-0.66,0.24)
3-dose: 70+	1.33 (0.84,1.82) *	-0.13 (-0.18, -0.08) *		-0.55 (-0.67, -0.44) *
3-dose (All)			2.36 (1,3.72) *	
Recovered	1.68 (1.48,1.87) *	0.79 (0.69,0.89) *	1.82 (1.26,2.39) *	0.29 (0.16,0.42) *
Recovered+ vaccine	1.03 (0.54,1.53) *	0.76 (0.64,0.89) *	1.18 (-0.85,3.21)	0.26 (0,0.51) *
**Age**				
12-15	0.04 (-0.06,0.13)	-0.16 (-0.24, -0.09) *		
16-39	ref.	ref.		
40-59	0.04 (-0.03,0.12)	0 (-0.05,0.04)		
60+	-0.17 (-0.29, -0.06) *	-0.65 (-0.72, -0.59) *		
**Sex**				
Female	ref.	ref.	ref.	ref.
Male	0.09 (0.03,0.15) *	-0.16 (-0.2, -0.12) *	0.1 (-0.09,0.29)	-0.14 (-0.21, -0.08) *
**Lab**				
Lab1	ref.	ref.	ref.	ref.
Lab2	0.99 (0.9,1.08) *	-0.77 (-0.97, -0.57) *	0.9 (0.6,1.2) *	-0.58 (-0.87, -0.29) *
Lab3	-0.57 (-0.65, -0.49) *	-0.3 (-0.35, -0.25) *	-0.43 (-0.7, -0.16) *	-0.72 (-0.8, -0.65) *
Lab4	-0.09 (-0.17,0) *	-0.73 (-0.79, -0.68) *	-0.49 (-0.77, -0.2) *	-0.81 (-0.91, -0.72) *
**R**	0.15 (-0.01,0.3)	0.79 (0.72,0.85) *		
**Calendar week**				
0			ref.	ref.
1			-0.05 (-0.29,0.19)	-0.11 (-0.25,0.02)
2			0.42 (0.15,0.69) *	-0.4 (-0.53, -0.27) *
3			0.7 (0.38,1.02) *	-0.62 (-0.75, -0.48) *
4			0.49 (0.11,0.88) *	-1.04 (-1.19, -0.89) *

Supplementary Table 5. Additional regression analyses. Analysis 1: Using reproduction number, R, instead of calendar time for both Delta and Omicron. Analysis 2: Truncation of follow-up study time and age in Delta, truncation of age in Omicron. 95% CI are given in parentheses. (*) denote 0.05 significance. Calendar time was partitioned to 1-week intervals.
