# Supplementary material for: Viral load dynamics of SARS-CoV-2 Delta and Omicron variants following multiple vaccine doses and previous infection
Source: Nat Commun. 2022 Nov 7;13:6706. doi: 10.1038/s41467-022-33096-0 (PMC9640564; doi:10.1038/s41467-022-33096-0)
Supplement: Supplementary file 2 — Reporting Summary [file 41467_2022_33096_MOESM2_ESM.pdf]

Corresponding author(s): Naama Kopelman

Last updated by author(s): Aug 3, 2022

Reporting Summary

Nature Portfolio wishes to improve the reproducibility of the work that we publish. This form provides structure for consistency and transparency in reporting. For further information on Nature Portfolio policies, see our Editorial Policies and the Editorial Policy Checklist.

Statistics

For all statistical analyses, confirm that the following items are present in the figure legend, table legend, main text, or Methods section.

n/a	Confirmed	
☐	☒	The exact sample size (*n*) for each experimental group/condition, given as a discrete number and unit of measurement
☐	☒	A statement on whether measurements were taken from distinct samples or whether the same sample was measured repeatedly
☒	☐	The statistical test(s) used AND whether they are one- or two-sided<br>*Only common tests should be described solely by name; describe more complex techniques in the Methods section.*
☐	☒	A description of all covariates tested
☐	☒	A description of any assumptions or corrections, such as tests of normality and adjustment for multiple comparisons
☐	☒	A full description of the statistical parameters including central tendency (e.g. means) or other basic estimates (e.g. regression coefficient) AND variation (e.g. standard deviation) or associated estimates of uncertainty (e.g. confidence intervals)
☒	☐	For null hypothesis testing, the test statistic (e.g. *F*, *t*, *r*) with confidence intervals, effect sizes, degrees of freedom and *P* value noted<br>*Give P values as exact values whenever suitable.*
☒	☐	For Bayesian analysis, information on the choice of priors and Markov chain Monte Carlo settings
☒	☐	For hierarchical and complex designs, identification of the appropriate level for tests and full reporting of outcomes
☒	☐	Estimates of effect sizes (e.g. Cohen's *d*, Pearson's *r*), indicating how they were calculated

Our web collection on statistics for biologists contains articles on many of the points above.

Software and code

Policy information about availability of computer code

Data collection	Software used to retrieve the data: SAS & Python 3
Data analysis	R (version 4.0.3) and R Studio (version 1.4.1103) were used for analysis. R code for producing the main figures and analyses is available at https://github.com/yonatan123/Viral-load

For manuscripts utilizing custom algorithms or software that are central to the research but not yet described in published literature, software must be made available to editors and reviewers. We strongly encourage code deposition in a community repository (e.g. GitHub). See the Nature Portfolio guidelines for submitting code & software for further information.

Data

Policy information about availability of data

All manuscripts must include a data availability statement. This statement should provide the following information, where applicable:

- Accession codes, unique identifiers, or web links for publicly available datasets
- A description of any restrictions on data availability
- For clinical datasets or third party data, please ensure that the statement adheres to our policy

The individual-level data cannot be publicly shared due to Israel Ministry of Health’s regulations. Requests for remote access to de-identified data should be referred to naamako@hit.ac.il, and will be assisted within 4 weeks pending IRB approval. Source data are provided with this paper.

Human research participants

Policy information about studies involving human research participants and Sex and Gender in Research.

Reporting on sex and gender	Sex was used as a covariate in regression analyses, since Ct differences between males and females are generally statistically significant. The assignment of sex was directly based on reported sex, as obtained from the dataset of the Israeli Ministry of Health.
Population characteristics	Infected individuals by vaccination status (age 5 years or above):<br>122416 unvacciantED (57015 male and 62717 female)<br>82981 2-dose (36188 male and 46597 female)<br>102347 3-dose (44140 male and 58002 female)<br>2565 4-dose (1366 male and 1199 female)<br>20443 recovered (9311 male and 11124 female)<br><br>Infected individuals by age, for the same vaccination statuses listed above:<br>5-11: 30456<br>12-15: 34181<br>16-39: 142475<br>40-59: 87655<br>60+: 35985
Recruitment	This is an observational study. Individuals included at this study were tested charge-free as part of the Israeli testing and surveillance program. Patient consent was waived, as this is a retrospective analysis using data which was collected as part of the national testing and surveillance program. The investigators did not have access to de-anonymized information.
Ethics oversight	Ethics. The study was approved by an Institutional Review Board (IRB) of the Sheba Medical Center. Helsinki approval number: SMC-8228-21. Individuals included at this study were tested charge-free as part of the Israeli testing and surveillance program. Patient consent was waived, as this is a retrospective analysis using data which was collected as part of the national testing and surveillance program. The investigators did not have access to de-anonymized information.

Note that full information on the approval of the study protocol must also be provided in the manuscript.

Field-specific reporting

Please select the one below that is the best fit for your research. If you are not sure, read the appropriate sections before making your selection.

- [x] Life sciences
- [ ] Behavioural & social sciences
- [ ] Ecological, evolutionary & environmental sciences

For a reference copy of the document with all sections, see nature.com/documents/nr-reporting-summary-flat.pdf

Life sciences study design

All studies must disclose on these points even when the disclosure is negative.

Sample size	Sample size was not restricted a-priori, but rather determined by the actual data in the Israeli Ministry of Health database.<br><br>Supplementary Table 1 reports sample sizes of ages 12 and above from each variant, within the date boundaries of the variants.<br>In addition, for each regression analysis and regression-based figure, the actual number of samples used in the analysis is stated in the legend. These numbers are simply the actual number of individuals used, after excluding individuals with missing data for one or more of the covariates, samples that are not between the defined date boundaries for Delta and Omicron, and samples that do not fit the defined cohorts for each analysis.<br><br>All bars in the figures (Fig. 1-3 & Supplementary Fig. 2-4) represent groups that consist of more than 50 observations each, with the exception of a single case: Lab2, Omicron variant, the 3-dose 10-39 days cohort; see Supplementary Table 1).
Data exclusions	Children under 12 were excluded from all of the main text analyses, since due to national policy individuals of ages 0-11 were not vaccinated until relatively late. Thus, ages 5-11 were only included in the sensitivity analysis, presented in the Supplementary Information section.<br><br>Individuals with unspecified age or sex were excluded from all regression analyses, since age and sex are used as covariates.<br>Measurements that do not fall between the defined date boundaries of each variant were excluded from analysis.

Repeated measurements of the same individuals were excluded if they are within 90 days from the same infection event. Only the first measurements was used from each group of such repeated measurements. Additionally. Ct measurements with more than 1 day separating the Ct date from the PCR sampling date were excluded from analysis, since we focus on early-stage viral load. Ct values<10 or >40 units were excluded since such values are likely the result of reading errors.

One-dose vaccinees were excluded from all of the analyses, since we focus on full vaccination statuses, alongside the Recovered.
The Recovered+vaccine cohort was excluded from analyses presented in the main text, in order to distinguish between natural infection based protection, and vaccine based protection.

Per analysis, individuals who do not fit any of the defined vaccination statuses were removed.

Replication: This is an observational study. However, we checked consistency across labs and genes.

Randomization: The allocation to cohorts is is relation to vaccination.

Blinding: Not relevant since this is an observational study.

Reporting for specific materials, systems and methods

We require information from authors about some types of materials, experimental systems and methods used in many studies. Here, indicate whether each material, system or method listed is relevant to your study. If you are not sure if a list item applies to your research, read the appropriate section before selecting a response.

Materials & experimental systems

n/a	Involved in the study	
☒	☐	Antibodies
☒	☐	Eukaryotic cell lines
☒	☐	Palaeontology and archaeology
☒	☐	Animals and other organisms
☒	☐	Clinical data
☒	☐	Dual use research of concern

Methods

n/a	Involved in the study	
☒	☐	ChIP-seq
☒	☐	Flow cytometry
☒	☐	MRI-based neuroimaging
